# Exercise Interventions for Women with Ovarian Cancer: A Realist Review

**DOI:** 10.3390/healthcare10040720

**Published:** 2022-04-13

**Authors:** Deirdre McGrath, Peter O’Halloran, Gillian Prue, Malcolm Brown, Joanne Millar, Adrina O’Donnell, Lisa McWilliams, Claire Murphy, Gwyneth Hinds, Joanne Reid

**Affiliations:** 1 School of Nursing & Midwifery, Queen’s University Belfast, Belfast BT7 1NN, UK; p.ohalloran@qub.ac.uk (P.O.); g.prue@qub.ac.uk (G.P.); m.brown@qub.ac.uk (M.B.); j.reid@qub.ac.uk (J.R.); 2 Belfast City Hospital, Belfast Health and Social Care Trust, Belfast BT9 7AB, UK; joanne.millar@belfasttrust.hscni.net (J.M.); drina.odonnell@belfasttrust.hscni.net (A.O.); lisa.mcwilliams@belfasttrust.hscni.net (L.M.); gwyneth.hinds@btinternet.com (G.H.); 3 Macmillan Cancer Support, Belfast BT9 7AB, UK; claire.murphy2@gll.org

**Keywords:** ovarian cancer, exercise intervention, implementation, realist review

## Abstract

Background: Despite evidence indicating the benefits of exercise interventions for women with ovarian cancer both during and following treatment, uptake is poor. There is limited research exploring the implementation of such interventions for this cohort of women. The purpose of this review was to identify implementation theories in relation to exercise interventions for women with stages I–IV ovarian cancer, both during and following treatment; to explain positive and negative contextual factors, which may help or hinder implementation; and to develop a theory on how exercise interventions for women with ovarian cancer may be implemented. Methods: This realist review sourced literature from five electronic databases: CINAHL plus, Medline, Embase, PsycINFO and Google Scholar. Methodological rigour was assessed using the relevant critical appraisal skills programme tools. Results: Nine papers were included. Two intervention stages were identified: first, optimising uptake by providing education to patients on the benefits of exercise, approaching patients when symptoms are adequately managed and offering a personalised exercise programme; second, adherence and retention are influenced by the provision of an “autoregulated” exercise programme with additional supportive infrastructure, individualised goal setting and symptom management support where required. Conclusion: Women with ovarian cancer are reluctant to engage in exercise interventions, despite the supporting evidence in terms of positive clinical outcomes. This realist review elucidates underlying mechanisms and important contextual factors that will support and guide the implementation of exercise interventions for this cohort of women.

## 1. Introduction

Ovarian cancer is the seventh most common cancer in women globally (World Ovarian Cancer Coalition). According to Globocan’s projections, by 2040, the number of women diagnosed with ovarian cancer will increase by almost 37% to over 428,000. Ovarian cancer is the leading cause of mortality among gynecological cancers in developed countries, with nearly 240,000 women diagnosed each year [1,2]. Previous studies advocated the importance of aerobic and resistance exercise programs for patients with a diagnosis of cancer [3,4]. Accumulating evidence suggests numerous health benefits can be obtained from engaging in regular exercise during and following treatment [5,6,7], including improvements in muscular strength, aerobic fitness [6], quality of life and physical function [6,7,8,9] and reductions in levels of depression and fatigue [6,8,9]. The American College of Sports Medicine recommends exercise programmes that incorporate moderate aerobic activity and muscle strength training both during treatment and into survivorship [10].

However, women both during and following treatment for ovarian cancer in stages I–IV of the disease are reluctant to engage in exercise and do not achieve the recommended exercise guidelines [11,12]. Current literature [2,9,11,12,13,14] reports wide variance in participation, adherence and retention. For the purpose of this review, it was necessary to operationalise these terms. Recruitment is the act of enlisting participants to take part in research [15]. Adherence is the proportion of planned intervention sessions that the participant completes [16]. Retention is retaining participants for the duration of the intervention period [17].

A realist review is a theory-driven approach that identifies how an intervention is supposed to work and then systematically gathers evidence to test and refine the theory [18] by critically examining the interaction between contexts (C), mechanisms (M) and outcomes (O) and the impact that these have on interventions (I) in a sample of identified studies [19,20,21]. Ultimately, it seeks to establish “what works, for whom and in what circumstances” [18]. A realist evaluation assumes that when any intervention is implemented, it changes the context within which it is implemented. The context is what is already present before the intervention is introduced, for example, the physical environment and the social structures [22]. The context also identifies the relationship between individuals and the process of implementing the intervention [23]. Mechanisms are the unseen factors, for example, a reduction in anxiety or increase in self-efficacy, that enable the implementation of the intervention and lead to its outcomes, such as behavior change [22,24]. Such issues have not been previously explored in relation to exercise interventions for women with ovarian cancer.

Previous literature reviews have identified positive effects of exercise interventions, such as improvements in clinical outcomes and physical functioning, women’s preferences in relation to the type of exercise and barriers to participation and adherence [5,10,25]. However, there is a paucity of literature that investigated the underlying theories that support the implementation of exercise interventions for this group of women, and the reasons for the lack of engagement have not yet been adequately explored [5,6,7].

To successfully implement an exercise intervention into practice, it is necessary to understand factors that motivate women to take part and sustain their engagement. Participation in exercise was shown to be influenced by self-efficacy [26,27], and it may be that the level of self-efficacy of women with ovarian cancer influences their decisions to engage with exercise interventions. This realist review was, therefore, undertaken in order to identify both the explicit and implicit theories underpinning exercise interventions, identify mechanisms of action and explain contexts that help or hinder the implementation of exercise interventions and participation by women with ovarian cancer (stages I–IV), both during and following treatment.

## 2. Review Questions

What components of exercise interventions facilitate or hinder their implementation and influence the participation of women with ovarian cancer?

What contextual factors help or hinder the implementation of the exercise interventions?

What mechanisms influence participation by women with ovarian cancer in exercise interventions?

## 3. Methods

This paper follows the RAMESES (Realist and Meta-Narrative Evidence Syntheses: Evolving Standards) guidelines for conducting and reporting the review [22].

### 3.1. Scoping the Literature

An initial exploratory literature search was undertaken, incorporating international research literature, grey literature and relevant policy documents, in order to understand broad approaches to implementation.

### 3.2. Information Sources and Search Strategy

The search strategy was developed in consultation with a subject librarian at Queen’s University Belfast. The databases searched included CINAHL plus, Medline, Embase, PsycINFO and Google Scholar (1980–March 2020). The reference lists of all articles included in this review were also searched, as was the British Library of E-Theses online (ETHOS) and Ovarian cancer charities websites, including Ovacome [28] and Target [29,30,31,32,33,34], to identify grey literature. The search strategy is presented in Table S1 (Supplemental File 1).

### 3.3. Selection and Appraisal of Articles

A defining feature of a realist review is the identification of the theoretical drivers for the intervention under review, in this case, exercise interventions for women with ovarian cancer. Articles were selected by focusing on their contribution to informing theory. Table 1 summaries the inclusion criteria for all papers in the review. We privileged the empirical literature for our synthesis, as it systematically reports clinical practice and outcomes. Methodological rigour was used to moderate the weights we gave to findings. The methodological quality of all included studies was assessed using the appropriate Critical Appraisals Skills Programme (CASP) tool [35] (Table 1).

### 3.4. Identifying Primary Sources

D.McG. reviewed each title and removed papers that were clearly not related to the subject or population of interest. Two reviewers (D. McG. and J.R.) reviewed each abstract and a consensus was reached about which papers to include for the full-text review. D.McG. reviewed all articles and each paper was independently reviewed by co-authors (J.R., P.O.H., G.P.), who each reviewed one-third of all papers.

### 3.5. Data Extraction

Data were extracted from the included full-text papers using a data extraction form used in previous realist reviews [29], which was based on the RAMESES guidelines for realist synthesis [19]. The form contains sections relevant to this realist review, including the theoretical background to the intervention, characteristics of participants and exercise interventions; how the intervention was thought to work; characteristics of implementation; and contextual issues that help/hinder implementation.

### 3.6. Identification of Candidate Theories

A realist review aims to identify the underlying programme theories and determine whether these theories are supported by the evidence [33]. A defining feature of a realist review is the identification of existing theories explaining how the intervention might work. These theories may be found in sources such as policy documents and the research literature [30]. Candidate theories were identified during preliminary scoping of the literature and were discussed and reviewed with all members of the research team.

### 3.7. Synthesis of Candidate Theories

The initial candidate theories in relation to exercise programmes for women with ovarian cancer, both during and following treatment, were developed further and synthesised during the process of reading and critically appraising each of the included papers. The components of the data extraction table from each study were synthesised and grouped into themes relating to the context (C), mechanism (M), outcome (O) and intervention (I). The candidate theories were reviewed in the light of these themes by all members of the research team and an inter-disciplinary advisory group who are experts in the delivery of care for women with ovarian cancer. The theories were combined in the theoretical model to indicate how and why exercise interventions for women with ovarian cancer may be successfully implemented in practice [33].

## 4. Results

### 4.1. Candidate Theories

The preliminary scoping of the literature did not identify any explicit theory underlying the implementation of exercise interventions for women with ovarian cancer. However, common sense implicit theories were identified. It was evident that women with ovarian cancer are reluctant to engage in exercise, despite clear benefits, both physically and psychologically [25,34,35,36]. If women with ovarian cancer are offered a structured and individualised intervention and the benefits in terms of well-being and possibly the reduced risk of recurrence are clearly explained to them, they are more likely to participate in an exercise intervention [37,38]. They are more likely to continue to engage in the exercise intervention if they receive support from those offering the service [2,9,11,12,36,39,40,41,42]. In addition, the level of self-efficacy of participants in exercise influences their decision to engage in exercise [27].

### 4.2. Study Selection

**Figure 1:**
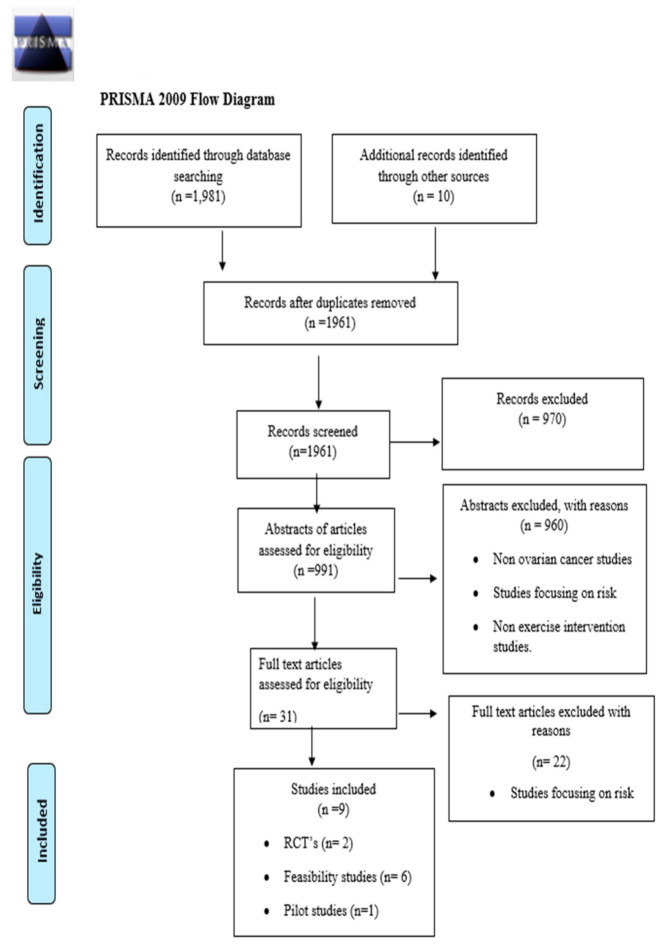
PRISMA 2009 flow diagram.

The search process identified 1991 records in total (Figure 1). The removal of duplicates left a total of 1961 papers. Nine hundred and ninety-one papers were selected for abstract review with reference to the inclusion criteria. D.McG. reviewed each title and removed papers that were clearly not related to the subject or population of interest. Two reviewers (D. McG. and J.R.) reviewed each abstract and a consensus was reached about which papers to include for the full-text review. D.McG. reviewed all full-text articles and each paper was independently reviewed by the co-authors (J.R., P.O.H., G.P.), who each reviewed one-third of all papers. This produced a total of 31 papers for the full-text review. Following the full-text review, 22 papers were excluded, leaving nine papers for data extraction. Reasons for exclusion are documented in Figure 1.

### 4.3. Study Designs

The nine empirical studies employed a range of designs. One was a pilot study with a quasi-experimental design [39], one was a feasibility study with a mixed-methods design [38], two were randomised controlled trials [11,12], four were feasibility studies with quantitative designs including three quasi-experimental designs [9,39,40] and one had a cross-sectional design [2].

### 4.4. Methodological Quality

The methodological quality of the empirical evidence in each of the nine papers included in this review were critically appraised using CASP [35]. All nine studies [2,7,8,9,36,37,38,39] were classified as moderate.

### 4.5. Main Objectives of the Studies

The included studies had two sets of objectives: the feasibility of implementing an exercise intervention in ovarian cancer populations [2,7,8,33,36,37,39] and the improvement of treatment-related symptoms for women with ovarian cancer either during or after treatment [9,11,12,39,40,41,42].

### 4.6. Study Populations

All studies in this review included women (*n* = 396) with ovarian cancer. The average age of participants was 59 years of age. The time points at which participants were recruited ranged from those who were six weeks to two years post-treatment [39,40,42] to those who were undergoing treatment, ranging from six weeks post-surgery to following the first cycle of chemotherapy treatment and throughout chemotherapy treatment [2,7,41,42]. Participants had diagnoses ranging from early to advanced disease. The majority of studies included women who had all stages (I–IV) of the disease [8,39,40,41,42]. The participants of two studies had a diagnosis of advanced disease, i.e., stages III and IV only 211. Four of the studies described recruiting participants from oncology units in the United States of America, two in Australia, one in China, one in Canada and one in Korea. It is notable that all studies were from high-income countries [43].

### 4.7. Characteristics of Exercise Interventions

All studies used multimodal techniques that involved implementing exercise and additional support. All studies involved some form of support throughout the intervention for the women, either in terms of regular telephone calls [29,42], group support [40,42], individualised in-person sessions [39,40], cognitive behavioral therapy [2,11] and education sessions regarding nutrition and symptom management [41]. In addition, differing approaches were used concerning the types of exercise incorporated, the setting and duration of the delivery of the intervention, the purpose of the intervention, techniques used to motivate participants and the techniques used to evaluate the intervention. The ensuing text will discuss the types of exercise programmes availed of, the settings in which these programmes were delivered, the techniques used to motivate participants and the outcomes.

### 4.8. The Types of Exercise Incorporated into the Intervention

Aerobics was the most common type of exercise and included either walking, swimming or cycling and required participants to increase their heart rate to a moderate level [8,9,39,41,42]. A combination of both aerobic and resistance exercises were also implemented [2,7,42]. Muscle stretching activity was also reported in one study [37].

### 4.9. The Context of the Delivery of the Interventions

The interventions incorporated home-based [2,8,9], supervised group setting [38] or a combination of supervised and home-based exercise sessions [4,36,39,40,41,42]. The ratio of the supervised and home-based sessions varied from one supervised to three home-based sessions weekly [9,42] to one supervised session for the initial six weeks of the study and then monthly [12]. One study required women to engage in aerobic exercise most days and incorporated a once-weekly supervised session [42].

### 4.10. The Outcomes of the Interventions

The studies measured the effectiveness of interventions in relation to levels of physical activity and patient clinical outcome measures, such as levels of fatigue, quality of life and levels of depression. Several studies reported significant improvements in patient outcomes, such as an increase in physical activity [39,40], cancer-related fatigue [9,11,39], health-related quality of life [9,11,36,40,41], depression [9,11,40] and aerobic fitness [2]. However, various tools were used to measure clinical outcomes. In relation to tools used to measure fatigue, studies included the Function Assessment of Cancer Therapy–General (FACT-F) [2,11,36,40], the Function Assessment of Cancer Therapy and the Somatic and Psychological Health Report [9]. In relation to depression, two tools were used: the Centre for Epidemiologic Studies Depression Scale (CES-D) scale [2] and the Hospital Anxiety and Depression Scale (HADS) [41]. Quality of life was measured using either the Function Assessment of Cancer Therapy–Ovarian (FACT-O) [2,41,44,45] or the Function Assessment of Cancer Therapy–General (FACT-G) [33,38].

Studies reported a wide variance in recruitment, adherence and retention rates. Participant recruitment varied from 16% [40] to 92% of those who were eligible to participate [41]. Retention rates varied from 26% [2] to 100% [43]. Participant adherence to interventions was primarily measured using activity diary/exercise logs [11,12,36,39] and electronic support, such as Fitbits and ACTi graphs [37,39]. Adherence ranged from 95% (Hwang et al., 2016) to 48% [39]. However, there was a lack of uniformity across all studies in relation to how adherence was defined. The exercise targets ranged from seventy-five minutes per week [39] to two hundred and twenty-five minutes per week [39]. In addition, some participants were required to reach one hundred percent of the target in order to be deemed as fully adhering [2,12,39,42], while others were deemed to have adhered if they achieved two-thirds of the target [36].

### 4.11. How the Intervention Was Thought to Work

None of the papers described an explicit theory underlying the implementation of exercise interventions for women with ovarian cancer. However, several implicit theories were identified relating to the method of recruitment, regular contact with participants, the shared group experience and an individualised approach.

Successfully recruiting participants is the initial step in implementing an exercise intervention [46]. In-person recruitment and indicating the potential benefits are effective recruitment methods [2,9,11,12,36,40,41]. In-person contact appears to stimulate the interest of potential participants and motivates them to take part [7,8,33]. Studies that used other recruitment methods, such as letters from the patient’s oncologist or information leaflets, failed to recruit as effectively [36,37].

Once recruited into a study, several articles proposed regular contact, either in person [33,36,37,38,39] or via telephone for both home-based and in-person programmes as methods to provide greater adherence [9,11,40]. Regular contact provides an opportunity to increase participants’ motivation by assessing whether participants are meeting the requirements of the intervention, such as completion of an exercise diary [9,36,39] or diet log [41], and providing positive feedback in relation to progress [29]. At the same time, those offering the intervention can identify barriers to participation, discuss possible solutions and provide education regarding management of physical symptoms [11,36,39]. The net result is to enhance the self-efficacy of participants and thereby increase adherence. This may lead to sustained behavioral change when participants continue to exercise post-intervention [9]. In a “virtuous circle”, exercise that results in positive clinical outcomes further rewards participants and motivates them to continue to engage in the intervention [36,39]. 

The shared experience evident in an exercise intervention delivered in a group environment enhances the self-efficacy of participants, thus increasing the motivation of women to adhere to the intervention [33,39]. The support and example of peers and the instructor are the principal influential factors that enhance the self-efficacy of participants regarding their ability to engage in the intervention [39,42]. Positive reinforcement and regular positive feedback are also influential factors. This is a result of the participant receiving direct guidance and reassurance from the instructor. Where the participant is observed engaging in the intervention, immediate feedback is given, thereby promoting engagement [39,42].

A flexible individualised approach empowers women to engage in the intervention and enhances their self-efficacy in relation to their ability to engage in the intervention. Achievable and personalised goal setting and time management are elements of this process [9,39,42], alongside identifying barriers to participation and discussion in relation to possible solutions [11,42] and providing participants with a choice in relation to the type of exercise they engage in [7,39,42]. In addition, the incorporation of additional support, such as regular contact with participants during the intervention [2,5,6,7,8,9,11,12], the addition of CBT [2,11] or education regarding diet and exercise [42], also enhances the self-efficacy of participants regarding participation. Therefore, an intervention that is flexible and accommodates the individual needs of each woman has a higher likelihood of women engaging fully in the intervention. All nine studies [2,9,11,12,42] included in this realist review are detailed in Table 2.

### 4.12. Contextual Factors That Help or Hinder Implementation of an Exercise Intervention for Women with Ovarian Cancer

Several contextual factors were identified that either help or hinder the implementation of exercise interventions for women with ovarian cancer. A clinical context in which exercise is seen by professionals as a core component of care will encourage participation in exercise programmes [2,5,6,7,8,9,10,11,12,36,39,40,41,42]. The mean ages of participants ranged from 53 years [2] to 60 years [43,44]. Younger participants may be more able to engage in an exercise intervention [2,12,40,41,42,43,44]. Having participants who were educated to a tertiary level (university or college education) was a common feature in the studies included in this review [2,41,42,43]. This may have resulted in increased awareness regarding the importance of exercise and increased adherence to the intervention. Women who have a pre-existing engagement in exercise (prior to their ovarian cancer diagnosis) were identified as being more inclined to participate in an exercise intervention [42]. In two studies, women post-aggressive treatment with improved physical function and a lack of treatment-related side effects were thought to be more likely to adhere to the intervention [12,44].

Contextual factors that hindered implementation were symptoms such as pain, fatigue and neuropathy, which discouraged adherence [9,12]; having to travel further distances to the class [41]; and schedule conflicts (where an exercise class was arranged at the same time as a clinical appointment) were also indicated as contextual factors that hindered this group of women from engaging in an exercise intervention [41]. A lack of interest in exercise in itself was also indicated as a reason for non-participation [9], with environmental conditions potentially impacting adherence to an exercise intervention [40].

### 4.13. Synthesis of Candidate Theories

**Figure 2:**
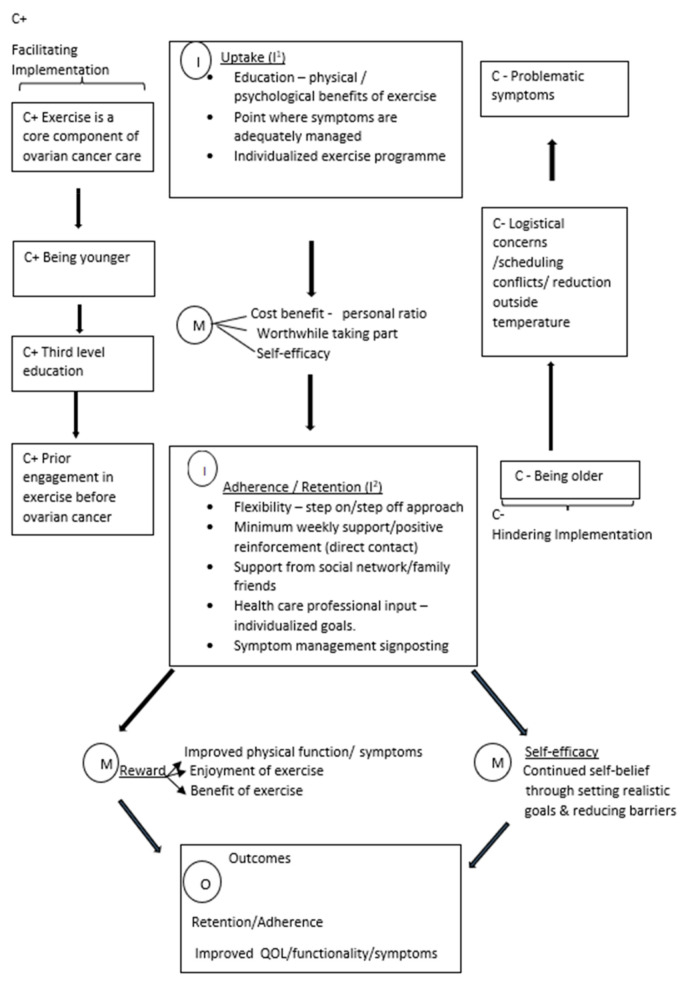
Theoretical model of how exercise interventions for women with ovarian cancer are thought to work.

In this section, the intervention (I), mechanisms (M) and contexts I that are thought to produce the outcome (O) of increased retention, adherence, quality of life, functionality and improvement in treatment-related symptoms are explained. The aim of this synthesis was to explain and support the future implementation of exercise interventions for women with ovarian cancer. The theoretical model of how exercise interventions for women with ovarian cancer are thought to work can be seen in Figure 2.

The implementation of exercise interventions involves two key phases: uptake (I^1^) and adherence/retention (I^2^). First, intervention uptake (I^1^) is dependent on several key factors: the healthcare professional who makes the first approach should do so in person, education should be provided to the potential participant in relation to the physical and psychological benefits of engaging in exercise, potential participants should be approached when their symptoms are adequately managed and the exercise programme should meet the individualised needs of participants. These factors help potential participants conclude that the personal cost–benefit ratio is worthwhile in terms of deciding to take part in the exercise intervention, where an in-person approach by a trusted professional communicates the high importance placed on the intervention, and the potential participant realises that the benefits are the educational material (M), whilst the individualised programme programe helps potential participants see that taking part could be feasible for them in terms of the costs in time and effort and its achievability, thus increasing self-efficacy in relation to the likelihood that they will succeed with the intervention (M).

Second, retention and adherence (I^2^) are dependent on several intervention characteristics. The intervention should be delivered with a flexible approach allowing participants to “step off” on days where they are feeling unable to participate and resume participation the following day (autoregulation); participants should be contacted regularly (at least weekly) to positively reinforce the progress made and to discuss actual and barriers to participation and to identify possible solutions. A healthcare professional should set individualised goals with participants and provide education in relation to how to manage symptoms. These intervention components work to reduce the costs experienced by participants by allowing flexibility when participation is challenging and addressing potential barriers whilst increasing intrinsic benefits by rewarding progress and recognising success (M). Continued adherence may then result in a positive feedback loop in that improvements in mental and physical well-being encourage continued engagement (M). Managing the costs in time and effort, addressing anxieties and difficulties and setting realistic goals with participants can also increase self-efficacy and, thus, promote adherence (M). These mechanisms are thought to result in enhanced retention and adherence to the exercise intervention and improvements in terms of quality of life, functionality and treatment-related symptoms (O).

Implementation may be hindered by several contextual issues, many of which seem to have their effect by increasing the cost of participation in terms of time and effort whilst reducing benefits. These include increased participant age and problematic symptoms, such as pain, neuropathy and fatigue; logistical concerns, such as travelling distance if the intervention is designed to be delivered in a specific venue; and scheduling conflicts when taking part in the exercise intervention clashes with clinical or other important appointments (C−). Implementation is more likely to succeed when it is routinely incorporated as a core component of care for women with ovarian cancer among younger age groups and among those who have a history of exercise prior to their diagnosis (C+).

## 5. Discussion

The initial candidate theory indicated in this realist review that was gleaned from a preliminary scoping of the literature indicated the importance of an individualised approach in delivering exercise programmes. Education regarding the benefits of exercise and the importance of continual support in encouraging adherence to exercise and the influence of self-efficacy on engagement in exercise were highlighted in the initial candidate theory [10,25,34,35,36,37,38,47]. This initial candidate theory was tested during the process of this realist review and is similar in some respects to the theoretical model presented in Figure 2, which was developed through identifying the underlying context, mechanisms and outcomes which lead to the successful implementation of an exercise intervention for women with ovarian cancer.

The theoretical model theorised that as women engage in exercise, the approach must be personalised and achievable [9,40,42]. An in-person approach to recruitment and a clear explanation of the benefits in terms of well-being results in an increased likelihood of participation [2,9,11,36,41,42]. Of note, these studies were conducted outside of the current COVID-19 pandemic and prior to the introduction of telephone reviews in response to the pandemic, which have become more common, thus reducing the number of face-to-face interactions between healthcare professionals and patients. The COVID-19 pandemic has heightened interest in using alternative approaches, such as telephone reviews to monitor and enhance the adherence of participants [47]. As women with ovarian cancer engage in exercise, they feel better physically [9,40,41,42], psychologically [2,9,42] and have enhanced functionality [42]. A multimodal approach incorporating additional components, such as regular telephone calls [12,39], CBT [2,8], education regarding symptom management, diet and exercise [39] has a positive effect on outcomes. A positive response to exercise, along with regular contact from study personnel, enhances the self-efficacy and quality of life of participants and they are more likely to fully engage in an exercise programme [2]. Due to the multi-modal approaches incorporated in the studies in this review, it is difficult to indicate the precise influential factors in terms of outcomes.

While several components were identified that either helped or hindered implementation, it is the interaction of context and mechanisms that may help to explain the successful implementation of the described exercise interventions. In order to encourage uptake of the intervention, exercise needs to be a core component of care. When clinical staff have an understanding and value the importance of engagement in exercise for women with ovarian cancer, this will increase the self-efficacy of potential participants and lead to them determining that it is worthwhile engaging in. Patients who are younger and have a tertiary-level education or have a history of prior engagement in exercise are predisposed to valuing and engaging in exercise and are more inclined to participate in and adhere to the exercise intervention. These positive contexts elicit particular mechanisms in relation to personal reward and increase in self-efficacy of participants, which leads to improvements in quality of life, treatment-related symptoms, physical functioning of participants and participation in and adherence to the intervention. Other factors can hinder implementation, such as participants experiencing problematic symptoms associated with treatment, being older, logistical concerns, scheduling conflicts and a reduction in outside temperature.

It is important to note that the studies included in this realist review are not representative of the typical demographic of women with ovarian cancer. Participants tended to be younger compared to the average age of women with ovarian cancer and have a prior interest in exercise. Each year, more than a quarter (28%) of all new ovarian cancer cases in the UK are diagnosed in females aged 75 and over [47]. Additionally, as is common in all research, lower educational levels may be indicative of decreased willingness to engage in research. This has implications in terms of how potential participants are approached and encouraged to engage in exercise interventions. The evidence reviewed excludes a core element of the demographic group. This anomaly may be due to the fact that people who are younger and more educated are more inclined to participate in research.

It is also important to note that the studies included in this review reported a wide variance in terms of participation, adherence and retention of participants. This variance does not appear to have been affected by the treatment stage of participants, which is in contrast to the wider literature in relation to exercise and cancer patients [3,4,47]. However, the authors of this review acknowledge that the treatment stage and type can affect the adherence and retention of participants in an exercise programme. All studies in this review incorporated some degree of supervision within the exercise programme, which was either conducted remotely via telephone, in person or a combination of both. However, the type of support does not appear to have affected the clinical outcomes or engagement of participants in the exercise programme. Therefore, the provision of support to participants appears to be the influential factor in terms of encouraging engagement in the exercise programme as opposed to whether the support is in person or offered remotely.

## 6. Strengths and Limitations

This is the first realist review to contribute to the development of a theoretical framework for the implementation of an exercise intervention for women with ovarian cancer, including the components necessary for the implementation and the underlying mechanisms that may contribute to outcomes. However, this review did not contain any realist evaluations and consisted of randomised controlled trials, a quasi-experimental study, a pilot study and feasibility studies, which provided important insight into underlying mechanisms but little guidance in relation to the explicit theories that underlie the successful implementation of exercise interventions in this patient population. Additionally, as exercise was implemented in combination with another intervention, it was difficult to identify which intervention resulted in the indicated outcomes. In addition, the research included in this review was from high-income countries only.

## 7. Conclusions and Recommendations

In conclusion, this realist review illustrated a theoretical framework that can inform the implementation of exercise interventions for women with ovarian cancer (stages I–IV), both during and following treatment. However, within the existing literature, there is a lack of a realist approach that explicitly theorises the specific contexts, components of the intervention and resulting mechanisms that result in the desired outcomes of improvements in treatment-related symptoms and enhanced participation, adherence and retention of these women to exercise interventions. This realist review elucidates underlying mechanisms and important contextual factors that can inform future realist evaluations. It is recommended that future research focuses on a realist approach to the implementation of exercise interventions for women with ovarian cancer.

Given the positive impact of exercise [2,5,6,7,8,9,10,11,12,36,37,38,39,40,41,42], greater efforts should be made to incorporate it as a core component of supportive care. Clinicians should assess their patients in relation to their exercise needs and provide appropriate advice regarding exercise or indeed refer them to suitably qualified exercise professionals and/or programmes [10]. A strategy involving an individualised approach to meeting exercise targets and sufficient behavioural support [9,40,42] may prove effective in ovarian cancer. Treatment-related toxicities and any other perceived barriers to participation should be addressed, with potential solutions to manage them identified. Presently, evidence regarding recruitment and engagement with exercise programming in women with ovarian cancer is extremely limited. Patient consultation, as part of a co-design approach, can assist in overcoming some of these issues and is thus recommended for future research that develops a successful implementation strategy.

## Figures and Tables

**Table 1:** Inclusion criteria.

Papers that were written in English. Papers that referred to women with ovarian cancer stages I–IV and all types of ovarian cancer. Papers published from 1980–March 2020. Empirical and non-empirical evidence. All types of study designs that implemented any type of exercise interventions (including aerobic, resistance, flexibility) with women with ovarian cancer during or following treatment.

**Table 2:** Summary of papers included in the realist literature review.

First Author (Year), Country and Objective	Population, Setting and Intervention	Design and Methodological Rigour (Strong/Moderate/Weak)	Key Results	How the Intervention Was Thought to Work	Contextual Factors Thought to Influence Implementation
Danhauer et al. (2008). North Carolina, USA. Objective: To engage participants in a 10 -week yoga programme to improve symptoms and treatment-related side effects.	Population: Women with ovarian (stages I, II, III, IV) or breast cancer—2 to 24 months post-primary treatment (surgery) following the initial diagnosis and/or had a recurrence of ovarian or breast cancer within the past 24 months. Participants were recruited during and following chemotherapy or radiotherapy treatment. Mean age—58.9 years. Convenience sample. Women with ovarian cancer (*n* = 37) (all stages of disease). Women with breast cancer (*n* = 14). Participants were recruited from two local cancer centres. Setting: group classes delivered in a structured environment. Intervention: The intervention consisted of 10 weekly 75 min once-a-week yoga classes taught by a yoga instructor certified by the National Yoga Alliance who had cancer-specific yoga training; The 10 class sessions combined physical postures breathing and deep relaxation; No home yoga practice was required.	Pilot/feasibility intervention study. Rigour: moderate. Small feasibility study. Single group design with no control group.	Recruitment rate: 16%. Retention/adherence rate: 60%. FACT-G—*p* = 0.05. Physical health and fatigue—*p* = 0.05. Qualitative feedback—88% of the class indicated they enjoyed the classes.	Not in-person recruitment was not as effective as in-person recruitment. The support and example of peers in the group and or the instructor may enhance self-efficacy, thus increasing motivation to adhere to the intervention. Enjoyment of intervention leads to enhanced adherence. Improvement in physical symptoms as intervention progresses motivates women to engage in the intervention. Flexible individualised approach empowers women to engage in the intervention.	The relatively young age (mean—59 years) of participants when compared to the average of ovarian cancer population may have enabled engagement and increased adherence. The majority of participants (89%) had tertiary-level education; this may have resulted in increased awareness regarding the importance of exercise and increased adherence to the intervention. Implementation of intervention hindered by: Distance to the venue of exercise class; Too busy/schedule conflicts; Health issues; Rarely (*n* = 3) the effects of cancer treatment.
Hwang et al. (2016). Seoul, Korea. Objective: To investigate the effects of a comprehensive care program on cardiopulmonary function, muscle strength, immune response and quality of life in ovarian cancer survivors.	Population: Ovarian cancer survivors with stages I [14], II [8] and III [18]; Post-surgery and chemotherapy; Participants in remission for 6 months to 3 years prior to enrolment; Participants selected did not exercise more than 30 min three times per week prior to recruitment; Experimental group *n* = 20; Control group *n* = 20. Setting: group sessions in a clinical environment and the exercise intervention was home-based. Intervention: 8-week program; Group education—one 40-min session per week for eight weeks; Self-help home-based exercise—three 50-min sessions per week for eight weeks: Brisk walking—30 min; CD—demonstrating exercises; Participants kept an exercise log; Participants were contacted twice weekly by study personnel to discuss their exercise routine. Relaxation—three 15 min sessions per week; Relaxation therapy was performed at home after the exercise sessions.	Quasi-experimental non-equivalent control group design. Weak: non-equivalent group design; small study and sample size.	Recruitment rate: 92% (self-help group support) and 95% (home-based exercise group). Retention rate: 80% (both groups). Adherence rate: 95% (self-help group support) and 91% (home-based exercise group). Cardiopulmonary function was measured using 12 min walk distance test (*p* = 0.05). Muscle strength measured using a chair to stand test—30 in total (*p* = 0.01). FACT-G experimental group—*p* = 0.003, *p* = 0.004, *p* = 0.001, *p* = 0.002.	The support and example of peers in the group and or the instructor may enhance self-efficacy, thus increasing motivation to adhere to the intervention. The twice-weekly telephone calls may have motivated participants to adhere to the intervention and to complete the exercise diary. Education—management of physical symptoms encourages participation, which is key to behavioural change. Combination of education, peer support and relaxation results in positive health outcomes. Choice in relation to the type of exercise enhances adherence.	The relatively young age (mean—60 years) of participants when compared to the average age of the ovarian cancer population may have enabled engagement and increased adherence. 55% of participants did not have a tertiary-level education; this does not appear to have affected the outcomes in terms of recruitment, adherence and retention of participants. Stage of treatment—participants were post-aggressive treatment; physical function improved post-treatment, lack of treatment-related side effects perhaps contributed to adherence. Previous interest in exercise enhanced motivation to participate.
Mizahi et al. (2015). Sydney and Canberra, Australia. Objectives: To determine the feasibility of a combined supervised and home-based exercise intervention during chemotherapy for women with recurrent ovarian cancer; To ascertain the impact of physical activity on psychological outcomes and chemotherapy completion rates.	Population: Women with recurrent ovarian cancer receiving second- or third-line chemotherapy (*n* = 30). Setting: home-based and one supervised session weekly. Intervention: 12-week exercise program; Individualised exercise program—90 min or more per week of low-to-moderate aerobic, resistance core stability and balance exercise; Aerobic resistance core stability and balance exercise sessions, 10–40 min duration, 3–4 times per week; Balance exercises—for those experiencing neuropathy and at risk of falls; Aerobic exercise—walking, cycling or swimming; Weekly telephone call to monitor adherence and progress and discuss program progression.	Prospective single-arm trial. Moderate—no explanation provided in relation to the statistical test used.	Recruitment rate: 55%. Retention rate: 70%. Adherence rate: 81%. Quality of life (*p* = 0.003). Fatigue (*p* = 0.004). Mental Health (*p* = 0.007). Muscular strength (*p* = 0.001). Balance (*p* = 0.003). 24 week follow up—participants maintained their physical activity levels.	Incorporation of behavioural change strategies (goal setting, time management, identification of methods to overcome barriers) into the intervention. Additional factors that enhanced adherence: Engaging participants in both aerobic and resistance training; Weekly telephone calls to monitor adherence and progress; Requesting participants to complete a self-report physical activity diary; Once weekly supervised session; Engagement in programme did not negatively affect the chemotherapy completion; Choice regarding the type of exercise enhanced adherence.	The relatively young age (mean—59 years) of participants when compared to the average of the ovarian cancer population may have enabled engagement and increased adherence. Due to advanced disease, 10% of participants withdrew from the study as a result of fatigue, pain and anxiety. Lack of interest indicated as a reason for non-participation. Treatment-related side effects negatively affected participation and adherence.
Moonsmammy et al. (2013). Toronto, Canada. Objectives: To assess the safety and feasibility of a walking and CBT intervention prior to and after the completion of chemotherapy; To improve musculoskeletal and cardiovascular fitness.	Population: Women with recently diagnosed ovarian carcinoma stages III or IV. Patients were recruited over four months. 46 eligible patients; 13 declined (felt unable to commit, lived too far away, felt they were already too active, involved in another exercise programme); Researchers were unable to contact 14 patients. N = 19: Treatment group *n* = 7; Surveillance group *n* = 12. Attrition rate 26%—2 drop-outs, 1 loss to follow up and 1 disease recurrence. Setting: home-based. Exercise and CBT intervention 24-week home-based exercise intervention. Co-ordinated with 12 weeks of CBT. Exercise intervention: Exercise program delivered by a certified exercise physiologist; Moderate aerobic exercise and resistance training; Participants completed aerobic and resistance training on alternate days and recorded weekly activity in a detailed exercise manual. CBT intervention: CBT counselling completed via phone; One hourly session every second week.	Non-randomised Phase 2 trial. Moderate—no justifications provided for design; Small sample size unable to generalise findings.	Recruitment rate: not available. Retention rate: 26%. Adherence rate: Surveillance phase—83%; Treatment phase—56%. Significant increase in aerobic fitness—in both treatment and surveillance groups (*p* = 0.08). Modest increases in HRQOL in both treatment and surveillance groups (not statistically significant). Higher levels of confidence and self-efficacy in the surveillance group (not statistically significant). Reduced levels of depression and post-traumatic stress (not statistically significant). Adherence to the exercise program was not indicated.	Fortnightly telephone calls may have contributed to adherence. The completion of the exercise diary may have also contributed to adherence. Improvements were noted in relation to the self-efficacy and motivation of participants—CBT may have influenced these factors.	The relatively young age of participants (mean–treatment phase—53 years, surveillance phase—58 years) when compared to the average of the ovarian cancer population may have enabled engagement and increased adherence. 58% of participants had a tertiary-level education; this may have resulted in increased awareness regarding the importance of exercise and increased adherence to the intervention.
Newton (2011). Brisbane, Australia. Objectives: To assess the safety feasibility and potential effect of a walking intervention in women undergoing chemotherapy for ovarian cancer; To assess whether engaging in physical activity resulted in participants completing their chemotherapy treatment and increased quality of life, reduced levels of depression and increased physical functioning.	Population: Women newly diagnosed with ovarian carcinoma stage I [1], stage II [1], stage III [11], and stage IV [4]. 27 eligible (reasons for non-participation were not identified). N = 17 participants receiving chemotherapy: 3 receiving neo-adjuvant chemotherapy; 14 receiving adjuvant chemotherapy. Sixty-two women presenting with possible ovarian cancer at the participating hospital were screened over approximately 15 months. Setting: delivered in a clinical environment and home-based. Intervention: Individualised walking programme delivered prior to and for the duration of chemotherapy treatment; Patients were provided with an information booklet regarding the walking intervention; Participants completed an activity logbook; Sessions with exercise physiologist once weekly (face to face or via telephone); Sedentary women were instructed to begin by walking frequently (most days) but with lower-intensity shorter-duration (10 min) walks; Active women were initially instructed to maintain their current number of sessions and first increase the duration and later the intensity; During weekly sessions with an exercise physiologist presence, changes to treatment-related side effects identified as barriers to walking were discussed and resolved when possible details of the previous week’s walking sessions were discussed, the subsequent week’s walking targets were discussed and participants were also asked to indicate how they felt about participating in the walking program; Post-intervention assessment was conducted three weeks after the last dose of chemotherapy.	Non-randomised Phase 2 trial. Moderate.	Recruitment rate: 63%. Retention rate: 100%. Adherence rate: 90%. Improvements in physical functioning *p* = 0.01. Improvements in FACT-O *p* = 0.01. Physical well-being *p* = 0.08. Ovarian-specific concerns *p* = 0.04. Sixteen women (94%) completed and returned the intervention evaluation, all of whom found the program to be either helpful or very helpful. The vast majority (81%) rated the sessions with the exercise physiologist as very helpful. 75% considered the program to be excellent.	Discussions with the exercise physiologist regarding barriers to participation (nausea and diarrhoea) and identifying solutions (modifying walking route). Setting individualised training goals with the exercise physiologist. Home-based intervention may have enhanced adherence as women were able to engage in the programme at a time that suited them. 1 weekly supervised exercise session enhanced adherence. Combination of exercise and cognitive behavioural therapy resulted in positive health outcomes.	The relatively young age (mean—60 years) of participants when compared to the average of the ovarian cancer population may have enabled engagement and increased adherence to the intervention. 71% of participants had a tertiary-level education, which may have resulted in increased awareness regarding the importance of exercise and increased adherence to the intervention.
Von Gruenigen et al. (2011). Objective: To assess the feasibility of a lifestyle intervention for promoting physical activity and diet quality during adjuvant chemotherapy for ovarian cancer.	Population: Women with ovarian, fallopian tube or peritoneal cancer (stages I, II, III, IV). Mean age—59 years. Setting: home-based and clinical environment. Intervention: Combination of home-based and in the chemotherapy clinic; Patients were enrolled post-operatively and received physical activity and nutrition counselling at every chemotherapy visit for six cycles; Patients enrolled prior to chemotherapy starting; This intervention was based on previous interventions in breast cancer (Pierce et al. 2002) and endometrial cancer patients (Von Gruenigen et al. 2008); Participants were seen individually at each chemotherapy session, either before or during infusion by the study’s registered dietician; Each individual session lasted 30 min; Participants were given guidance specific to the intake of nutrient-dense foods and staying as physically active as possible; Participants were given pedometers and asked to complete a daily exercise and diet log; Participants were asked to be as physically active as possible; Questionnaires were completed at the beginning of each chemotherapy session.	Prospective single group trial of a nutrition and physical activity intervention in patients receiving at least 6 cycles of adjuvant chemotherapy. Moderate—small sample size.	Recruitment rate: 73%. Retention rate: not available. Adherence rate: 92%. Increase in physical activity cycle 3 to following cycle 6 was 61 min (*p* = 0.03) in cycle 3 to was 73 min (*p* = 0.082) in cycle 6. Increase in FACT-G *p* = 0.001. MSAS score *p* = 0.01. Increased moderate to strenuous physical activity was correlated with higher physical well-being during chemotherapy *p* = 0.037.	Setting of individualised goals may have increased adherence as participants had achievable targets. Discussing progress weekly enhanced adherence. The completion of daily activity logs and wearing pedometers. The authors theorised that increased physical activity may increase the quality of life (Stevinson et al. 2007) and the ability to tolerate chemotherapy and improve survival. Regular physical activity may increase the quality of life and reduce levels of fatigue and, subsequently, the ability to respond to and tolerate chemotherapy was enhanced. Participants were usually accompanied by a family member to the one-on-one sessions, therefore enhancing the participants’ motivation to adhere to the intervention.	The relatively young age (mean—59 years) of participants when compared to the average of the ovarian cancer population may have may have enabled engagement and increased adherence. 66% of participants had a tertiary-level education, which may have resulted in increased awareness regarding the importance of exercise and increased adherence to the intervention.
Zhang et al. (2018). China. Objective: To examine the feasibility of a nurse-led home-based exercise and cognitive behavioural therapy for ovarian cancer adults with cancer-related fatigue on outcomes of fatigue, sleep disturbance and depression.	Population: Ovarian cancer patients with stages I (*n* = 2), II (*n* = 10), III [33] and IV [21]; Recently post-surgery following a diagnosis of ovarian cancer; Patients with moderate [4,5,6] to severe [7,8,9,10] levels of fatigue (National Comprehensive Cancer Network 2011). Recruited to trial following the first cycle of adjuvant chemotherapy. Participants (*n* = 72): Randomly assigned to experimental group (*n* = 36) or comparison group (*n* = 36). Setting: 12-week home-based CBT and exercise intervention. Exercise intervention: Exercise—Walking or cycling; Patients were provided with an exercise manual and required to engage in aerobic physical exercise three to five times per week for twenty-five to sixty minutes per session; Aerobic and resistance training; Physical exercise 3–5 times per week for 25–60 min per session was recommended; The intensity of the exercise was gradually increased over the course of the intervention; Participants kept an exercise log; Weekly telephone contact from the research nurse to provide information regarding fatigue and to monitor exercise methods. CBT intervention: CBT intervention—delivered via internet sessions once weekly, 1 h each session for 12 consecutive weeks.	Randomised controlled trial. Moderate.	Recruitment rate: 92%. Retention rate: 92%. Adherence rate: T1—83.2%, T2—76.1%, T3—73.7%. Patients were deemed to adhere if they completed a minimum of 3 sessions per week for a 25 min duration per session. Fatigue: T1—Experimental group had statistically significant levels of behavioural (*p* < 0.001), sensory (*p* < 0.007) and cognitive (*p* < 0.036) fatigue; T2—no statistically significant reduction was noted in relation to affective fatigue; T3—Patient’s had statistically significant levels of behavioural (*p* < 0.001), sensory (*p* < 0.001) and cognitive (*p* < 0.001) fatigue. Depression: T2 and T3—experimental groups had statistically significant lower levels of depression (*p* ≤ 0.001). Strengths: The research nurses provided counselling regarding fatigue information, which may have helped with adherence to exercise intervention.	The use of the weekly telephone calls to monitor adherence to the exercise interventions and to provide motivational support may have positively impacted adherence to the intervention. Participants had an exercise manual to provide information in relation to the exercise intervention. Participants were required to complete an exercise diary. The research nurses provided counselling regarding fatigue information, which might have helped patients engage in physical activity intervention at home. Participants wore a Fitbit for the duration of the study.	The relatively young age (42% 55 years or younger) of the participants compared to the average of the ovarian cancer population may have may have enabled engagement and increased adherence.
Zhou et al. (2017). USA. Objective: Examine the effect of a six-month aerobic exercise intervention vs. attention control on the change in HRQOL and CRF in women diagnosed with ovarian cancer.	Population: 1-year post-diagnosis—diagnosed with ovarian cancer within last four years at the time of randomisation); All stages—I (*n* = 34) II (*n* = 30), III [5,8] and IV [21]; 54% of participants had stage III or stage IV cancer. Participants (*n* = 144)—mean age 57.3 + 8.6 years. Setting: home-based. Intervention: Exercise arm: Six-month home-based moderate-intensity aerobic exercise program (mainly brisk walking); 150 min of moderate-intensity aerobic exercise—mainly brisk walking; Weekly telephone calls to each participant from an American college of sports medicine certified cancer exercise trainer; Using a weekly 26-chapter book that the researchers developed; The trainer provided weekly individualised counselling via telephone to motivate participants to exercise; Seven-day activity log was used as the primary exercise adherence measure; Women wore heart monitors and were given a targeted heart range based on the Karvonen method for moderate-to-vigorous intensity; Participants recorded their exercise and heart rate in their daily activity log andreported this information to the exercise trainer during the weekly phone calls. Attention control arm: Received a weekly phone call from a WALC staff member, along with a 26-chapter book that contained ovarian cancer survivorship-related information.	Randomised controlled trial. Moderate.	Recruitment rate: not available. Retention rate: 78%. Adherence rate: Exercise—65%; Phone calls: 25 calls—19% (control arm) 47.3% (exercise arm); 20 calls—73.5% (control arm) 79.9% (exercise arm). Statistically significant improvement in the FACT-F (*p* < 0.02). Participants in the exercise arm who adhered to 80% or more of telephone calls had statistically significant improvement in FACT-F (*p* = 0.04). Participants in the attention control arm who adhered to 80% or more of the telephone did not have a statically significant improvement in FACT-F.	Motivational support via weekly telephone calls (boosted with 26-chapter book that contained motivational support) enhanced adherence. The additional supports, i.e., heart rate monitor and exercise diary, also assisted in motivating participants to adhere to the intervention. Weekly telephone calls focusing on participants’ adherence.	The relatively young age (mean age 55 years) of participants compared to the average of the ovarian cancer population may have enabled engagement and increased adherence. 39% of participants in the exercise arm had a tertiary-level education, which may have resulted in increased awareness regarding the importance of exercise and increased adherence to the intervention. 23% in the experimental arm had disease recurrence during intervention, which affected adherence.
Zhang et al. (2017). USA Objective: To establish the feasibility and acceptability of a 26-week home-based high dose exercise intervention among women with advanced-stage ovarian cancer.	Population: 10 women with advanced ovarian cancer at stages III and IV. Setting: home-based. Intervention: 26-week home-based high-dose exercise intervention of 225 min per week—moderate-intensity, mainly walking; All participants were provided with a walking program DVD and a physical activity tracker at their first in-person session; In-person exercise sessions were provided weekly for the first six weeks and monthly for the remaining 20 weeks by a certified clinical exercise trainer; Weekly telephone contact by a research staff member based on an interview script for the entire 26 weeks; Participants were asked each week whether they had any symptoms that prevented exercise; Participants were asked to keep an exercise log for the duration of the intervention; Participants wore an ACTi graph accelerometer for one week prior to the intervention and during the final week; Participants were asked to document in a diary provided accelerometer wear time.	Non-randomised. Pre-/post-test pilot study.	Recruitment rate: 50%. Retention rate: 80%. Adherence rate: 80%. During the 26 weeks, moderate-intensity exercise increased by 15 min per day (*p* = 0.05). The barriers to exercise indicated by participants were pain, neuropathy, lymphedema, life events stress and vacation. The mean number of steps per day increased from 948 to 1162.	In-person exercise sessions were facilitated by a certified exercise trainer. Individualised exercise plans. Participants wore a Fitbit for the duration of the study and an activity tracker for the first and final weeks of the study. Participants were asked weekly to indicate barriers that may have prevented them from exercising during the previous week.	The relatively young age (mean age 55 years) of participants compared to the average of the ovarian cancer population may have enabled engagement and increased adherence. Mean time since diagnosis—44 months, which may have resulted in positively impacting adherence as participants were more able to engage in the intervention. Temperature outside affected adherence—an increase in physical activity was associated with an increase in temperature.

Explanation of abbreviated terms: FACT-G—Functional Assessment of Cancer Therapy—General; HRQOL—health-related quality of life; FACT-F—Functional Assessment of Cancer Therapy—Fatigue; MSAS score—Memorial Symptom Assessment Scale; DVD—digital video disc; CBT—cognitive behavioural therapy.

## Data Availability

Not applicable.

## Supplementary Materials

The following supporting information can be downloaded at: https://www.mdpi.com/article/10.3390/healthcare10040720/s1, Table S1: Search strategy—Included as supplemental file.
