# Supplementary material for: Exercise Interventions for Women with Ovarian Cancer: A Realist Review
Source: Healthcare (Basel). 2022 Apr 13;10(4):720. doi: 10.3390/healthcare10040720 (PMC9024745; doi:10.3390/healthcare10040720)
Supplement: Supplementary file 1 [file healthcare-10-00720-s001.zip › healthcare-1609209-supplementary.pdf]

healthcare

MDPI

Supplemental file

Table S1. Search strategy—Included as supplemental file.

	Search term
1	Ovarian neoplasms /or ovarian carcinoma
2	Ovarian cancer or ovarian neoplasms
3	Gynaecologic cancer (mp)
4	Gynaecologic carcinoma (mp)
5	Gynaecologic neoplasms
6	Ovarian carcinoma (mp)
7	1 or 2 or 3 or 4 or 5 or 6
8	Physical activity (mp) or exercise
9	Movement or movement (mp)
10	Aerobic exercise (mp) or exercise
11	Rehabilitation
12	Joint mobility
13	Ambulation or walking
14	Exercise therapy or therapeutic exercise (mp)
15	Muscle control (mp)
16	Balance (mp)
17	8 or 9 or 10 or 11 or 12 or 13 or 14 or 15 or 16
	7 and 17

Key words and MSH terms: CINAHL plus, Medline, Embase, PsycINFO.
